# The role of tumour heterogeneity and clonal cooperativity in metastasis, immune evasion and clinical outcome

**DOI:** 10.1186/s12916-017-0900-y

**Published:** 2017-07-18

**Authors:** Deborah R. Caswell, Charles Swanton

**Affiliations:** 1 Translational Cancer Therapeutics Laboratory, The Francis Crick Institute, 1 Midland Rd, London, NW1 1AT UK; 2 Cancer Research UK Lung Cancer Centre of Excellence, University College London Cancer Institute, Paul O’Gorman Building, 72 Huntley Street, London, WC1E 6BT UK

**Keywords:** Intratumour heterogeneity, Tumour progression, Metastasis, Linear evolution, Branched evolution, Competitive evolution, Cooperative evolution, Mutation burden, Immunotherapy, Aneuploidy tolerance

## Abstract

**Background:**

The advent of rapid and inexpensive sequencing technology allows scientists to decipher heterogeneity within primary tumours, between primary and metastatic sites, and between metastases. Charting the evolutionary history of individual tumours has revealed drivers of tumour heterogeneity and highlighted its impact on therapeutic outcomes.

**Discussion:**

Scientists are using improved sequencing technologies to characterise and address the challenge of tumour heterogeneity, which is a major cause of resistance to therapy and relapse. Heterogeneity may fuel metastasis through the selection of rare, aggressive, somatically altered cells. However, extreme levels of chromosomal instability, which contribute to intratumour heterogeneity, are associated with improved patient outcomes, suggesting a delicate balance between high and low levels of genome instability.

**Conclusions:**

We review evidence that intratumour heterogeneity influences tumour evolution, including metastasis, drug resistance, and the immune response. We discuss the prevalence of tumour heterogeneity, and how it can be initiated and sustained by external and internal forces. Understanding tumour evolution and metastasis could yield novel therapies that leverage the immune system to control emerging tumour neo-antigens.

## Background

In his 1958 essay, Foulds [1] explains that linear tumour progression, the orthodox view of tumour evolution at the time, is the theory that neoplasia advances through an orderly sequence from local invasion, to progressive lymph node invasion, to metastasis [1]. Foulds goes on to discuss tumour progression and metastasis in several cancer types and concludes that neoplasia is not linear, but a complex, ever-diverging route throughout tumour development [1]. This was one of the first articulate explanations of tumourigenesis as a multistep pathway that can progress, persist, or regress [1]. Nowell’s 1976 [2] cancer evolution model proposed that genomic instability drives branched evolutionary pathways from a clone of origin. Heppner [3] reviewed literature on the emergence of heterogeneity in tumours, and the challenges surrounding its study, emphasising that tumour cells exist in a society, and that the interactions between heterogeneous populations are as important as the subclones themselves [3].

Recent technological advances have allowed scientists to study tumour complexity in more detail. Following exome sequencing on multiple spatially separated samples obtained from primary carcinomas and metastatic sites, intratumour heterogeneity and parallel evolution of subclonal driver events was uncovered [4–7]. Multi-region sequencing has now been performed in many cancer types including breast, lung, colorectal, renal, oesophageal cancer and glioma, uncovering intratumour heterogeneity in all cancer types studied [4–15].

## Drivers of heterogeneity

Intratumoral heterogeneity exists in many forms, from somatic coding and non-coding alterations to epigenetic, transcriptomic and post-translational modifications [16, 17]. Intratumoral copy number heterogeneity also exists (see [16]). Many endogenous triggers of cancer genome instability contribute to intercellular heterogeneity (see [18]), some of which may be therapeutically exploitable. Defective DNA mismatch repair results in hypermutation and microsatellite instability, and mutations inhibiting the proofreading ability of DNA polymerases δ and ε increase base mismatches [19]. Evidence also exists that tumour cell dormancy contributes to tumour heterogeneity (see [20]). Recently, it has been uncovered that APOBEC (apolipoprotein B mRNA editing enzyme catalytic polypeptide) family members are endogenous drivers of tumour diversity in many tumour types [21]. These enzymes initiate DNA cytosine deamination [21, 22], and are a major source of subclonal cancer gene mutations in bladder, breast, head and neck squamous cancers, lung adenocarcinomas and lung squamous cell carcinomas [21, 23–26]. External factors such as cytotoxic therapy [27, 28], and patient factors such as genetic background [29, 30], can also influence tumour heterogeneity.

## Mechanisms mediating levels of genomic instability and tumour heterogeneity

Cahill and Vogelstein [31] discussed the conflict between disadvantages and advantages of genomic instability in tumour evolution. They questioned how cancer cells are able to select for alterations driving genomic instability, which is, usually disadvantageous to the cell, can sometimes lead to cell death, and has no direct growth advantage [31]. Looking to basic studies of mutation rate and cellular fitness in bacteria, the authors reasoned that in stressful environments bacteria with higher overall levels of genomic instability eventually dominate the population because they can adapt [31]. This model can be applied to tumour populations, where genomic instability may be critical for tumour progression [31].

Most normal diploid cells negatively select against chromosomal instability (CIN). This is partially mediated by p53, which inhibits cell propagation after genome instability [32, 33]. CIN mouse models support the concept that low or moderate CfIN levels promote tumour formation, but excessive CIN suppresses tumour formation [34]. This is analogous to mutational meltdown and error-prone catastrophe in bacterial and viral genetics [34–37]. Aneuploid, specifically trisomic cell lines, grow poorly in vitro and as xenografts compared to genetically matched euploid cells [38]. Yet, following prolonged growth, aneuploid cells adapt by acquiring additional alterations correlating with improved fitness [38]. While aneuploidy was detrimental initially, over time it became more advantageous.

In tumours, selection might favour the mitigation of excessive CIN to prevent cell autonomous lethality. Partial dysfunction of anaphase-promoting complex/cyclosome (APC/C) lengthens mitosis, allowing more time for correction of impending chromosome segregation errors. This permits tumour cells to fine-tune CIN during tumour evolution, navigating the delicate equilibrium between cell death as a result of too much or too little genomic instability [39]. In colorectal cancer, alterations in *BCL9L* promote tolerance of chromosome missegregation events, propagation of aneuploidy and genetic heterogeneity [40]. This tolerance is induced because BCL9L dysfunction leads to lower levels of caspase-2, impairing MDM2 cleavage, p53 stabilisation, and generation of the pro-apoptotic protein tBID, upon chromosome missegregation [40].

Once cells have undergone genome doubling (GD), propagation of aneuploidy follows as the tolerance of GD allows cells to endure continuing CIN [41]. One mechanism of GD tolerance involves cyclin D1, which overrides a p53/p21-dependent checkpoint in G1, allowing tetraploid cells to proliferate [42, 43]. Additional mechanisms mediating levels of instability include buffering protein changes caused by aneuploidy, activating autophagy, and enhancing proteasomal degradation [44, 45]. Mechanisms that both buffer and allow cells to tolerate instability contribute to heterogeneity in tumours, as cells that propagate genomic alterations and aneuploidy survive. Understanding the processes that balance tumour heterogeneity and promote aneuploidy tolerance may contribute to novel therapeutics to prevent tumour heterogeneity and drug resistance.

## Tumour heterogeneity and metastasis

The study of intratumour heterogeneity has yielded novel findings about the timing and evolutionary drivers of metastasis. Metastasis is a multistep process consisting of local invasion, intravasation, survival in the circulation, extravasation and distant colonisation [46, 47].

**Fig. 1:**
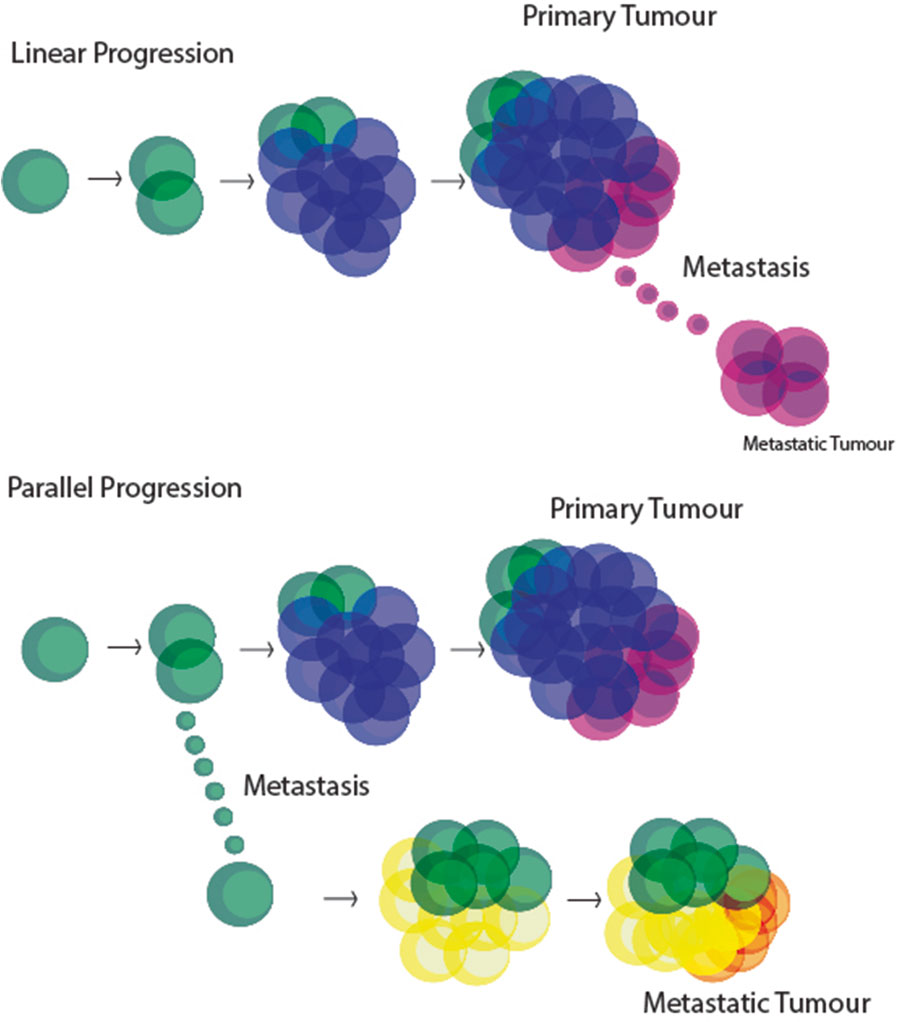
The linear progression model versus the parallel progression model of tumour evolution. In the linear progression model (*upper panel*), late stage tumour cells disseminate and form metastases. In contrast, in the parallel progression model (*lower panel*), early tumour cells disseminate and form metastases alongside the primary tumour, and both primary tumour and metastases progress in parallel gaining multiple subclonal populations

Two prominent models of metastasis are the linear and parallel progression models [46–48]. Both are founded on the clonal relationships between a primary tumour and its metastases [47]. In the linear progression model, metastasis is seeded at a late stage of tumour progression, resulting in minimal genetic divergence between the primary tumour and its metastases [47]. Conversely, in the parallel progression model, metastases are seeded early in tumour progression, so high levels of genetic divergence are expected between the primary tumour and its metastases [47, 49, 50] (Fig. 1). To reconcile either model requires accounting for the subclonal complexity of the primary tumour and its relationship to subclones present at the metastatic sites (see [47]).

In colorectal, breast, pancreatic, renal, and prostate cancer, multiregional sequencing supports both metastatic models [47]. Both models were observed in one colorectal cancer case [51]. One large metastasis had genetically diverged from the primary tumour suggesting early formation, while the other metastases were similar to the primary tumour, so most likely disseminated at a late stage of tumour progression [51]. This implies that heterogeneous subclones can form multiple distinct metastases. In breast and pancreatic cancer mouse models, metastases aligned with the parallel progression model [50, 52–54], whereas the linear progression model was supported in a lung adenocarcinoma mouse model [48].

These data suggest metastasis is complex and diverse, and that tumour heterogeneity is critical in the metastatic process. Since more work is required to understand tumour heterogeneity in late stage tumours and metastases, in depth efforts are now examining post-mortem tumour heterogeneity. The UK national PEACE autopsy (posthumous evaluation of advanced cancer environment) program, may help to decipher the complexity cancer evolutionary processes at death.

## Subclonal interactions in heterogeneous tumours

### Background and *Drosophila melanogaster* models of subclonal interaction

It was recognised in the 1970s that heterogeneous subclones within tumours contain differing capacities for growth and metastatic ability [55–57]. Early work also recognised that interactions between subpopulations resulted in differences in drug sensitivity when injected into opposite flanks of mice or co-cultured in collagen [58, 59]. The importance of these studies was not fully recognised until over two decades later [60].

Growing interest in tumour heterogeneity and subclonal interactions led to the development of a useful model for studying subclonal competition and cooperation in *Drosophila melanogaster* [60]. Eichenlaub et al. [61] used a *D. melanogaster* model of epithelial tumour formation to show that overexpression of both epidermal growth factor receptor (EGFR) and miR-8 in wing imaginal disc cells results in supercompetitive cells that engulf those surrounding them. These supercompetitive cells drive tumourigenesis and metastasis, whereas cells overexpressing either EGFR or miR-8 alone do not. Competition between normal and oncogenic tissues was also uncovered: in *D. melanogaster*, imaginal epithelial cells activate nonapoptotic JNK signalling in response to oncogenic mutant cells [62]. This leads to the activation of the ELMO/Mbc-mediated phagocytic pathway, which eliminates oncogenic cells [62]. Growing evidence suggests that subclones within tumours compete, but also cooperate. Several *D. melanogaster* studies demonstrated cooperation between cells with oncogenic *Ras* ^*V12*^ mutations and cells lacking the scribbled gene *(scrib-/-)* to promote growth and invasion of the *Ras* ^*V12*^ mutant cells [60, 63].

### Xenotransplant models of subclonal interaction

Xenotransplant models have been used to understand interactions between human tumour subclones. In a glioblastoma multiforme mouse xenotransplant model, a minor mutant EGFR subpopulation enhanced tumourigenicity of the entire tumour. This occurred via a paracrine mechanism that promoted growth in wtEGFR cells within the tumour, illustrating the advantages of heterogeneity [64]. In a zebrafish-melanoma xenograft model, inherently invasive (MITF^high^) melanoma cells cooperated with poorly invasive (MITF^low^) cells to invade away from the primary site through solid tissue [65]. The protease activity and extracellular matrix deposition of MITF^high^ cells around the primary tumour was critical for this co-invasion of MITF^high^ and MITF^low^ cells [65]. Heterogeneity may be critical in tumour progression and metastasis.

Polyak and colleagues [66] used a mouse xenograft model to investigate the effect of a polyclonal tumour population on tumour growth and metastasis. A minor IL11-overexpressing subclone changed the tumour microenvironment by increasing intratumoural vascularisation and reorganising extracellular matrix, in turn increasing tumour growth [66]. Importantly, this subclone was also outcompeted by a faster proliferating competitor, leading to tumour collapse [66].

These xenograft studies provide insights into subclonal interaction dynamics, demonstrating their complexity, and that they are involved in many aspects of tumour evolution including tumorigenesis, vascularisation, invasion and metastasis.

### Mouse models of subclonal interaction

Advances in molecular biology have allowed a deeper study of subclonal cooperation in cancer mouse models. Autochthonous models permit long-term study of clonal dynamics in a specific genetic context [67].

In a mouse model of small cell lung cancer (SCLC), tumours were composed of distinct cell populations with either neuroendocrine or mesenchymal markers [68]. Both cell types shared specific genomic aberrations derived from a common ancestor [68]. Transition between the neuroendocrine and mesenchymal phenotype was achieved through ectopic expression of Ras^*V12*^, and the mixed population endowed mesenchymal cells with metastatic ability [68].

Cleary and colleagues [69] used a mouse model of breast cancer to show that interclonal cooperation can be critical for tumour propagation. A portion of tumours harboured distinct *Hras* ^*mut*^ *Wnt* ^*low*^ and luminal *Hras* ^*wt*^ *Wnt1* ^*high*^ subclones, which cooperated to maintain tumour propagation [69]. Although Wnt activation is rare in human breast cancer, this remains a valuable model for studying paracrine mechanisms of subclonal communication.

In a p53-null mouse model of basal-like breast cancer, two tumour cell populations were identified, one expressing mesenchymal markers (“mesenchymal-like” cells), and another defined as tumour-initiating cells (TICs) [70]. When both populations were co-transplanted using limited dilutions, mesenchymal-like cells promoted self-renewal and tumour initiation capacity of the TICs [67]. Surprisingly, the mesenchymal population was maintained as a minor subpopulation [70].

Mouse models have revealed how subpopulations within tumours cooperate to promote tumour growth, maintenance and metastasis. Although not fully representative of the clinical presentation of disease in patients, these autochthonous models show the diverse ways in which subclones can interact and affect tumour progression. These studies suggest that to more effectively treat heterogeneous tumours, we must first understand the dynamics between different populations within a tumour, and how targeted treatment changes these interactions.

### Therapy and subclonal resistance

Therapeutic resistance often develops in the advanced setting following therapeutic targeting of clonal alterations, as subclones containing somatic events driving resistance can pre-exist or be generated *de novo*.

In non-small cell lung cancer (NSCLC) a low frequency subclone with MET amplification was selected for during treatment with EGFR tyrosine kinase inhibitors (TKI), allowing for development of a resistant MET- amplified tumour [71]. The EGFR T790M mutation, which drives EGFR TKI resistance, was also present prior to treatment in NSCLC [72]. In colorectal cancer, EGFR antibody-resistant subclones with pre-existing KRAS mutations emerged in 38% of patients following 5–6 months of treatment with panitumumab [73]. In patients with NSCLC, a subset of EGFR TKI resistant cancers acquire alterations, such as RB and p53 loss contributing to small cell carcinoma transformation [74].

In patients with HER2-positive breast cancer, Janiszewska et al. [75] discovered a dramatic increase in a minor resistant population of PIK3CA mutant cells, and a slight decrease in the dominant population of HER2-amplified cells post neoadjuvant therapy with trastuzamab. Alterations in the phosphoinositide 3-kinase (PI3K)-AKT pathway commonly drive resistance in patients treated with the HER2-targeting antibody trastuzamab [75].

In patients with colorectal cancer, mutant *KRAS* clones emerging during treatment with EGFR-specific antibodies declined during treatment breaks [76]. Engelman and colleagues [77] demonstrated that resistance to EGFR TKIs can develop through pre-existing clones and newly developed drug-tolerant clones simultaneously, though through separate mechanisms. When treating the NSCLC cell line PC-9 with increasing concentrations of gefitinib, one resistant population derived from EGFR T790M pre-existing clones emerged early, while another with characteristics of a drug-tolerant state and the EGFR T790M mutation, emerged late. This late emergent EGFR T790M subpopulation arose from drug-tolerant cells that were, initially, partially resistant to gefitinib, and then gained the EGFR T790 mutation to become fully resistant [77].

These studies demonstrate that heterogeneous tumours develop resistance when specific clones are targeted, allowing pre-existing or newly evolved subclones to emerge.

Competition between subclones is frequently revealed during and following therapy exposure. Keats and collaborators [78] illustrated that, in a patient with multiple myeloma, clonal dominance alternated between two main subclones. Modelling these subclones in a mouse, treatment with bortezomib exerted selective pressure on all subclones, but one clone eventually emerged and outcompeted the others [78]. Conversely, minor therapy-resistant subpopulations can also support the survival of therapy-sensitive populations, preserving tumour heterogeneity. In patients with colorectal cancer, EGFR therapy-resistant KRAS mutant subclones support non-mutant therapy-sensitive cells by secreting increased levels of TGFalpha and amphiregulin [79], in turn sustaining EGFR/ERK signalling in sensitive cells [79]. These data demonstrate how resistance to targeted therapy can develop with both preservation and loss of heterogeneity.

### Heterogeneity and patient outcome

Is intratumour heterogeneity associated with worse patient outcomes? In premalignant Barrett’s oesophagus, clonal diversity is an important predictor of tumour progression [80]. By adapting diversity measures from ecology to measure both the number and abundance of clones relative to others in the population, the upper quartile of the number of clones and the genetic divergence based on loss of heterozygosity (LOH) were strongly predictive of increased progression from Barrett’s oesophagus to oesophageal adenocarcinoma [80].

In breast, ovarian, gastric and NSCLC, there is a paradoxical relationship between CIN and prognosis. The worst outcomes are in tumours with intermediate CIN, while those with extreme CIN scores had an improved outcome [81, 82]. The tumour cell of origin and the order of somatic events may also influence tumour heterogeneity and patient outcome [83].

In a recent study of 12 different cancer types [84], mortality risk increased when more than two clones coexisted in the same tumour sample, but decreased with the coexistence of more than four clones, emphasising that heterogeneity levels within tumours can strongly influence outcome [84]. Tumours with high levels of somatic copy number alterations (SCNAs) have increased proliferation markers and a decreased immune signature [85]. Interestingly, both phenotypes are controlled by different types of aneuploidy, and may be important patient outcome markers, as patients with higher SCNA levels tend to have worse responses to immunotherapy [85]. Recently, our group demonstrated in the TRACERx prospective study of 100 patients with NSCLC, that heterogeneity of DNA copy number events rather than point mutations, was associated with poor outcome [86].

In breast cancer, enriched areas of tandem duplications previously thought to be unimportant passengers, were shown to be potentially important for tumourigenesis [87]. These tandem duplications were characterised into two different rearrangement signatures [87]: one enriched in areas that disrupted tumour suppressor genes such as *PTEN* and *RB1*, and the other enriched in oncogenes, including *MYC*, and in putative regulatory elements of genes such as *ESR1*, with direct effects on transcription [87]. These enrichments demonstrate that mutational processes not only stochastically mutate the genome and induce heterogeneity but are also likely to be critical in cancer evolution and patient outcome. Further work to understand how established clinical parameters of outcome such as tumour size, grade and stage reflect intratumour heterogeneity is required.

### Heterogeneity and immune responses in cancer

Emerging evidence suggests that intratumour heterogeneity may also influence the anti-tumour immune response. Rapid advances in cancer genome sequencing have enabled scientists to decipher the impact of somatic coding alterations upon immune recognition and surveillance. Non-silent mutations generate neoantigens that can be recognised by the immune system [88, 89]. Both CD4+ and CD8+ T lymphocytes were shown to recognise tumour neoantigens [89–93]. Using whole genome sequencing, scientists have established that mutation burden is highly variable among different tumour types [94]. Mutation levels in melanomas and lung cancers are among the highest because of exposure to ultraviolet light and tobacco carcinogens respectively [94, 95]. This makes these cancer types ideal candidates for immunotherapy; however, others such as clear cell renal cancer also respond to checkpoint therapy despite a much lower mutational burden [96].

### Mutation burden and immunotherapy

In patients with melanoma, whole exomes from pre-treatment melanoma tumour biopsies and matching germline tissue samples were examined to determine if neoantigen burden affects responses to antibodies directed against cytotoxic T lymphocyte-associated antigen-4 (CTLA4) [97]. Overall mutational load and expression of cytolytic markers in the immune microenvironment were significantly associated with an anti-CTLA4 response and clinical benefit [97].

Rizvi and colleagues [98] explored how mutational burden affects sensitivity to PD-1 blockade in NSCLC. Expressed by activated T cells, PD-1 is a key immune checkpoint receptor that mediates immunosuppression [88]. They concluded that a higher nonsynonymous mutation burden was associated with improved objective response, durable clinical benefit and progression-free survival [98]. However, some patients with both high and low mutational burdens failed to respond to therapy [98], suggesting that other factors are involved in immunotherapy responses.

We investigated whether melanoma and NSCLC sensitivity to PD-1 and CTLA-4 treatment was enhanced in tumours where tumours had a high clonal neoantigen burden. Considering a combination of neoantigen clonality and neoantigen burden allowed us to better discriminate responder from non-responder patients than either metric alone [99]. These findings suggest that the degree of intratumour heterogeneity may be associated with a differential response to checkpoint blockade, and that multiple factors are involved in tumour immune responses. These studies raise the possibility that technologies now exist to exploit clonal neoantigens, present in every tumour cell for therapeutic benefit, either through vaccination or cell therapy approaches.

### Resistance and immune evasion

Multiple laboratories are now focusing on a deeper understanding of immune evasion and resistance to immunotherapy. One study [100] uncovered two novel mechanisms of evasion: first, the elimination of neoantigen-containing tumour cells within a subset of the tumour population, and second, the acquisition of one or more genetic events that resulted in neoantigen loss. Both clonal and subclonal alterations were lost following immunotherapy, but all clonal neoantigens were eliminated by chromosomal deletions and LOH, whereas subclonal neoantigens were lost through LOH and elimination of tumour cells [100]. We uncovered that CIN likely contributes to mutational heterogeneity within tumours through SCNAs [86]. More than 14% of subclonal mutations (range: 0–56%) are subclonal because of SCNA loss events of clonal alterations [86]. This suggests that CIN contributes to neoantigen heterogeneity, and may play a role in immune evasion and immunotherapy resistance. These findings raise questions about whether immunotherapy will be effective as tumours evolve and become heterogeneous, and to what extent underlying CIN, resulting in rapid karyotype evolution, drives resistance to such approaches. Understanding neoantigen heterogeneity within tumours will aid the development of more effective, patient-specific immunotherapies, which may not prevent eventual relapse but could significantly extend patient’s lives.

T cell exhaustion is another mechanism of immune evasion. This state of T cell dysfunction arises during chronic antigen exposure, which is defined by poor effector function, sustained expression of inhibitory receptors, and an abnormal transcriptional state [101–103]. In this state, PD-1 is one of many cell surface inhibitory receptors to co-regulate T cell exhaustion [103]. Anti-PD-1 immunotherapy partially restores T cell function from an exhausted state [102, 104, 105]. It is also clear that the relative abundance of partially exhausted tumour-infiltrating CD8+ T cells predicts response to anti-PD-1 therapy [106]. However, resistance to PD-1 therapy develops in many patients.

Alterations in the genes encoding interferon receptor-associated Janus kinase 1 (JAK1) or 2 (JAK2), along with deletion of the wild-type allele, lead to defects in pathways involved in interferon receptor signalling and in antigen presentation. This results in resistance to PD-1 blockade in melanoma [107]. Loss of PTEN also inhibits T cell-mediated tumour-killing by decreasing T cell infiltration in tumours, and is associated with worse outcomes with anti PD-1 therapy [108]. These studies suggest that tumour heterogeneity is beneficial to immune evasion and the development of immunotherapy resistance. Approaches that attenuate tumour heterogeneity by inhibiting the molecular mechanisms driving cell-to-cell variation, or that target multiple truncal mutations occurring consistently in all regions of the tumour while stimulating the immune response using checkpoint blockade, might help limit diversity and resistance acquisition [99].

## Conclusions

We have begun to witness how tumour heterogeneity affects tumour progression and metastasis, and the tumour immune response. A deeper understanding of how the immune system can be leveraged to tackle clonal alterations within tumour cells is required, to identify high-risk tumour subclones that might be extinguished prior to, or at the time of, metastatic seeding. Longitudinal characterisation of tumour evolution using advanced sequencing technology is important, as will be characterisation of the distinct mechanisms of tumour heterogeneity from tumour diagnosis through to progression and death. These studies may open avenues to attenuate tumour heterogeneity in minimal residual disease when disease burden is low, or to target multiple clonal mutations present in every cell simultaneously to limit the acquisition of therapy resistance.

As tumour heterogeneity is arguably the major force behind tumour progression, evolution and metastasis, insight into the clonal complexity of individual tumours and its contribution to progression, immune evasion and exhaustion may be critical to the development of more effective cancer therapeutics.
